# Announcement of 2019 Keystone Symposia Conference: “Microbiome: Chemical Mechanisms and Biological Consequences”

**DOI:** 10.1128/mSystems.00115-18

**Published:** 2018-07-24

**Authors:** Emily P. Balskus, Peter J. Turnbaugh, Dennis W. Wolan

**Affiliations:** a Department of Chemistry & Chemical Biology, Harvard University, Cambridge, Massachusetts, USA; b Department of Microbiology & Immunology, University of California San Francisco, San Francisco, California, USA; c Chan Zuckerberg Biohub, San Francisco, California, USA; d Department of Molecular Medicine, The Scripps Research Institute, La Jolla, California, USA; e Department of Structural and Computational Biology, The Scripps Research Institute, La Jolla, California, USA

**Keywords:** biology, chemistry, Keystone conference, microbiome

## Abstract

The Keystone Symposia will be hosting a conference organized by Emily Balskus, Peter Turnbaugh, and Dennis Wolan entitled “Microbiome: Chemical Mechanisms and Biological Consequences” 10 to 14 March 2019 in Montreal, Québec, Canada. Our goal for this meeting is to focus attention on the intersection of chemistry and biology by bringing together scientists in these two disciplines, while also including talks about other hosts, environmental microbiomes, and multidisciplinary research platforms. The focus of this conference is to emphasize our community’s need to continue adopting other scientific disciplines to ultimately generate a broad understanding of microbiomes and the cross talk microbes have with their environment. We are inviting speakers from across the globe that interrogate fundamental chemical processes of microbiomes, including small-molecule and xenobiotic metabolism, natural product synthesis, and the many microbial enzymes responsible for the production of these biologically relevant metabolites. The ability to link the chemical foundations of microbes with biological outcomes would provide tremendous contributions to this emerging field of study.

## Abstract (precis)

The Keystone Symposia will be hosting a conference organized by Emily Balskus, Peter Turnbaugh, and Dennis Wolan entitled “Microbiome: Chemical Mechanisms and Biological Consequences” 10 to 14 March 2019 in Montreal, Québec, Canada. Our goal for this meeting is to focus attention on the intersection of chemistry and biology by bringing together scientists in these two disciplines, while also including talks about other hosts, environmental microbiomes, and multidisciplinary research platforms.

## EDITORIAL

Microbiome research has primarily focused on human health and has stemmed from biomedical and ecological research methodologies. While sequencing-based techniques combined with gnotobiotic mouse models, newly cultured bacterial strains, and overall advances in microbiology have provided exceptional insights into microbial communities, elucidation of fundamental molecular mechanisms and the chemical languages behind the complex interplay between microbes and their environment have lagged behind. Similarly, the emphasis on human-related microbiomes needs to be expanded to promote more research on environmental microbiomes. Broadening the scope of research and including chemical biological, pharmacological, and biochemical research initiatives into the microbiome at a molecular level would have far-reaching consequences in basic biology, human health and disease, and therapeutic design.

Our goal for this meeting is to spotlight innovative research at the intersection of chemistry and biology as well as present key research initiatives on other hosts, environmental microbiomes, and multidisciplinary research platforms. There is a critical lack of microbiome meetings with this primary goal, and such programs are needed to bring together scientists broadly interested in these areas of research and address fundamental questions and provide forums for brainstorming and discussions about the future of microbiome research. The collection of speakers and attendees are interested in hypothesis-driven research that will help us better understand microbial ecology at a molecular and chemical level. Along with speaker sessions, each day will have workshops targeted toward students and fellows that will highlight emerging chemical biological techniques, the role of biotech and pharmaceutical companies in microbiome therapeutics, and insights into host-microbiome and microbe-microbe interactions. The conference will conclude with a general discussion and brainstorming session on future directions of microbiome research.

The program for this Keystone Symposia meeting will begin with a plenary lecture by Jon Clardy from Harvard University and include the following sessions: (i) microbiome biochemistry; (ii) small molecules in host-microbiome cross talk; (iii) microbiome toxicology and pharmacology; (iv) antibiotics; (v) enzyme discovery; (vi) emerging technologies; (vii) natural products; and (viii) synthetic and systems microbial ecology. For a full list of speakers, please visit http://www.keystonesymposia.org/19C3. We urge graduate students and postdoctoral fellows to apply for conference scholarships provided by the Keystone Symposia, and the deadline for applications is 8 November 2018. The discounted abstract and regular abstract deadlines are 8 November 2018 and 6 December 2018, respectively, for attendees wishing to present their recent research findings, and each session will consist of several short talks chosen from the abstracts. The deadline for registration with discounted fees is 16 January 2019.

We anticipate the presentations, workshops, and poster sessions on cutting-edge technologies, multidisciplinary research, and transformative findings will promote active and lively discussions among the academic and industry microbiome-focused attendees and encourage the establishment of cross-disciplinary research programs and collaborations at the interface of chemistry and biology. We hope to see you and your associated microbiomes soon in Québec, Canada.

